# Evidence of a chimeric genome in the cyanobacterial ancestor of plastids

**DOI:** 10.1186/1471-2148-8-117

**Published:** 2008-04-23

**Authors:** Jeferson Gross, Jörg Meurer, Debashish Bhattacharya

**Affiliations:** 1 University of Iowa, Department of Biological Sciences and the Roy J. Carver Center for Comparative Genomics, 446 Biology Building, Iowa City, Iowa 52242, USA; 2 Department Biology I, Botany, Ludwig-Maximilians-University Munich, Menzinger Str. 67, 80638 Munich, Germany

## Abstract

**Background:**

Horizontal gene transfer (HGT) is a vexing fact of life for microbial phylogeneticists. Given the substantial rates of HGT observed in modern-day bacterial chromosomes, it is envisaged that ancient prokaryotic genomes must have been similarly chimeric. But where can one find an ancient prokaryotic genome that has maintained its ancestral condition to address this issue? An excellent candidate is the cyanobacterial endosymbiont that was harnessed over a billion years ago by a heterotrophic protist, giving rise to the plastid. Genetic remnants of the endosymbiont are still preserved in plastids as a highly reduced chromosome encoding 54 – 264 genes. These data provide an ideal target to assess genome chimericism in an ancient cyanobacterial lineage.

**Results:**

Here we demonstrate that the origin of the plastid-encoded gene cluster for menaquinone/phylloquinone biosynthesis in the extremophilic red algae Cyanidiales contradicts a cyanobacterial genealogy. These genes are relics of an ancestral cluster related to homologs in Chlorobi/Gammaproteobacteria that we hypothesize was established by HGT in the progenitor of plastids, thus providing a 'footprint' of genome chimericism in ancient cyanobacteria. In addition to *men*B, four components of the original gene cluster (*men*F, *men*D, *men*C, and *men*H) are now encoded in the nuclear genome of the majority of non-Cyanidiales algae and plants as the unique tetra-gene fusion named *PHYLLO*. These genes are monophyletic in Plantae and chromalveolates, indicating that loci introduced by HGT into the ancestral cyanobacterium were moved over time into the host nucleus.

**Conclusion:**

Our study provides unambiguous evidence for the existence of genome chimericism in ancient cyanobacteria. In addition we show genes that originated via HGT in the cyanobacterial ancestor of the plastid made their way to the host nucleus via endosymbiotic gene transfer (EGT).

## Background

Bacterial chromosomes are subject to a constant influx of genes through horizontal gene transfer (HGT) that often occurs from phylogenetically distant organisms [1]. When foreign genes confer a selective advantage they are maintained by the host organism and vertically transmitted to descendents. Consequently, over evolutionary time the continual introduction of foreign genes into bacterial chromosomes is expected to result in a highly chimeric genome. Estimates of the frequency of HGT in prokaryotes are variable depending on the bioinformatic approach used, the organism(s) analysed, and inherent limits to the detection of intraphylum HGT among closely related taxa [1-4]. Overall, percentages of genes acquired by HGT in a typical prokaryote tend to average 2–25% of the total genome [1-4] but exceptional cases are known with values as low as 1.6% in *Mycoplasma genitalium *or as high as 32.6% in *Treponema pallidum *[1]. Regardless of the actual number that is cited, the magnitude of observed HGTs demonstrates that a large fraction of genes in extant prokaryotes originated via lateral transfer. The question remains open whether HGT might have had a similar, or even more dramatic impact on early bacterial evolution. The genomes of ancient prokaryotes could potentially differ substantially in terms of gene content and phylogenetic diversity from their present-day counterparts [4,5]. These considerations are particularly intriguing when formulated in the context of the endosymbiotic origin of the plastid that is derived from the capture of a cyanobacterium ca. 1.5 billion of years ago [6,7]. This endosymbiotic occurred in the founding lineage of photosynthetic eukaryotes, the Plantae [8]. Here we ask the question, is there evidence of chimericism in plastid genes that would provide insights into ancient cyanobacterial genomes? The plastid is a 'living fossil' ideally suited for this purpose because it has largely been protected from HGT within the eukaryotic host [9] (for exceptions see below), thereby conserving its ancient genome characteristics.

The plastid genome is a highly reduced version of the cyanobacterial endosymbiont [8], retaining between 54–264 genes [10]. Phylogenetic surveys of conserved plastid genes convincingly demonstrate their cyanobacterial ancestry [11,12]. Furthermore, phylogenomic analyses indicate that ca. 500–1500 cyanobacterial genes were transferred to the nucleus (endosymbiotic gene transfer, EGT) and their gene products are largely targeted back to the plastid [13-15]. Three known cases contradict the notion of a strictly cyanobacterial ancestry for plastid genes. The first two are the *rbc*L gene in red algae and chromalveolates and the *rpl*36 gene shared uniquely by cryptophytes and haptophytes that are phylogenetically most closely related to homologs within the Alphaproteobacteria and to a Planctomycete related to *Rhodopirellula baltica*, respectively [9,16]. The third case is a firmicutederived gene for the tau/gamma subunit of DNA polymerase III (*dnaX*) encoded in the plastid genome of the cryptophyte *Rhodomonas salina *[17]. *DnaX *is absent from all other sequenced plastid genomes including that of another cryptophyte (*Guillardia theta*) [17]. Their limited distribution (*rpl36*, *dnaX*) and nested relationship to Alphaproteobacteria (*rbc*L) suggest that these HGT events are unlikely to be examples of genome chimericism that was vertically inherited from the captured cyanobacterium followed by differential loss in Plantae [9,16,17]. In contrast, here we provide phylogenetic evidence that plastid and nuclear-encoded proteins for the biosynthesis of phylloquinone (PhQ), a redox cofactor of the Photosystem I complex in algae and plants, may represent surviving traces of a chimeric genome in the captured endosymbiont.

## Results

### Homologous genes for PhQ biosynthesis are plastid-encoded in Cyanidiales but nuclear-encoded in the majority of photosynthetic eukaryotes

**Figure 1:**
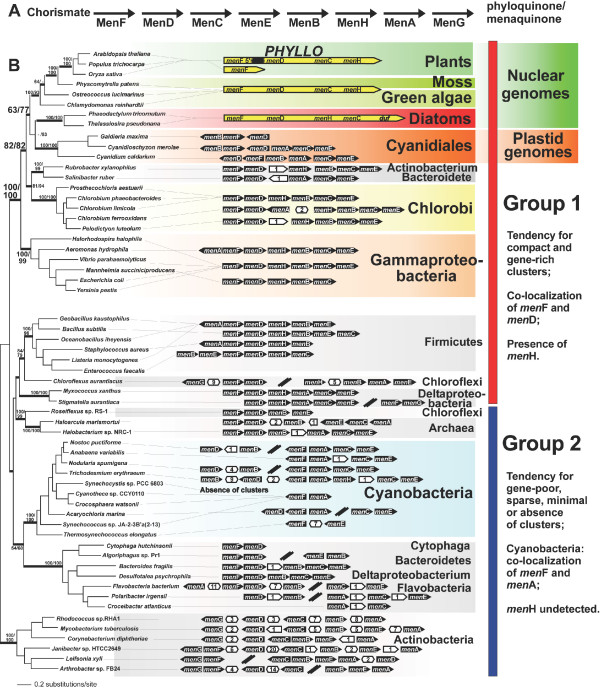
**PhQ/MQ methabolic pathway and phylogeny of MenFD proteins.(a) **Shown are the Men proteins that control the enzymatic steps necessary for the conversion of chorismate to the final product MQ in bacteria and PhQ in cyanobacteria and in photosynthetic eukaryotes. **(b) **Bayesian majority rule consensus tree of a concatenated alignment of MenF and MenD proteins. The nodal numbers represent bootstrap support values inferred using PHYML (left of slash mark) and neighbor joining (1000 replicates, right of slash mark). Only bootstrap values >50% are shown. For the sake of clarity only support values at nodes of highest interest have been included in the figure. The thick branches have a BPP > 0.95. The branch lengths in this tree are proportional to the number of substitutions per site (see scale in figure). *Men *genes encoded as clusters in chromosomes of prokaryotes and in the plastid genome of Cyanidiales (black boxes), as well as the architecture of the *PHYLLO *gene (yellow boxes) in nuclear genomes of photosynthetic eukaryotes, are indicated for each taxon. The break in the 5' terminus of *PHYLLO *in higher plants indicates a gene-splitting event during evolution [18]. In addition, in plants *men*F is an individual gene distinct from *PHYLLO*. The *men *gene cluster in the plastid of *Galdieria maxima *was only partially sequenced. White boxes with numbers inside indicate the number of genes with functions unrelated to the menaquinone biosynthesis that separate the *men *genes. Double slashes indicate a large chromosomal separation between *men *genes. According to their structure, gene clusters can be divided in two groups (Group 1 and Group 2) that are correlated with the tree topology. This tree was arbitrarily rooted on the branch leading to the Actinobacteria.

Biosynthesis of the Photosystem I cofactor PhQ occurs in cyanobacteria, plants, and algae and is analogous to that of menaquinone (MQ), a mobile electron carrier in many bacterial bioenergetic systems [18]. Both biosynthetic pathways have been characterized at the genetic and biochemical level in prokaryotes and eukaryotes and depend on 8 enzymatic steps catalyzed by the so-called 'Men proteins' (Figure 1a) [18-23]. In MQ-using bacteria and cyanobacteria, *men *homologs are present as individual genes usually grouped within operons or in proximally encoded transcripts (Figure 1b). In available genomes of plants, green algae, and stramenopiles *men*A, *men*B, *men*E, and *men*G are nuclear-encoded and constitute independent loci. Genes encoding MenF, MenD, MenC, and MenH tend to be united (with some rearrangements) as a single nuclear-encoded, composite gene termed *PHYLLO *(Figure 1b and Materials and methods) [18]. In higher plants *men*F (*isochorismate synthase*, *ICS*) is an independent nuclear-encoded gene that is distal to *PHYLLO *(Figure 1b) [18].

In contrast to their nuclear location in most algae and plants, *men *genes are plastid-encoded in the early diverging Cyanidiales red algae *Cyanidium caldarium *and *Cyanidioschyzon merolae *(Figure 1b). In these taxa, the *men *homologs (including *men*F, *men*D, and *men*C that are fused in nuclear encoded *PHYLLO*) are present as individual genes and grouped in a compact cluster that resembles the structure of prokaryotic operons. The nucleotide sequence of the plastid *men *cluster of the Cyanidiales alga *Galdieria maxima *has been partially determined in this study and displays a similar prokaryoticlike genetic organization that appears to be syntenic with the locus in *Cyanidioschyzon merolae *(Figure 1b). Collectively, these observations lead us to postulate that plastid and nuclear-encoded *men *genes in algae and plants are of endosymbiont origin and the nuclear location of most genes is due to EGT.

### *Men*F, *men*D, *men*C, *men*B, *men*E, and *men*H were originated via a single HGT event in the ancestor of plastids

To test the hypothesis of a common endosymbiotic origin of algal and plant *men *genes, we first inferred separate maximum likelihood phylogenies for MenF and MenD. These trees were essentially congruent (results not shown) therefore we concatenated the proteins to gain greater phylogenetic resolution. To generate the alignment, we used the two proteins that are already fused in PHYLLO in algae, whereas in plants we concatenated MenF (i.e., the ICS1 paralog in *Arabidopsis*) with MenD in PHYLLO. The resulting MenFD tree (Figure 1b) recovers the monophyly of PHYLLO and MenFD from the Cyanidiales (Bayesian posterior probability, BPP = 0.98, PHYML bootstrap, PB = 3%, neighbor joining bootstrap, NJB = 77%), consistent with the idea that the tetra-fusion *PHYLLO *arose from an ancestral plastid gene cluster whose remnants are still present in the plastid genome of Cyanidiales. The distribution of operon characteristics (not their overall phylogeny) supports the existence of two superclades that we provisionally named Group 1 and Group 2. Group 1 contains compact, gene-rich *men *gene clusters, the co-localization of *men*F and *men*D, and contains *men*H, whereas Group 2 is defined by relatively gene-poor, dispersed (as in cyanobacteria) clusters. Group 1 contains the unexpected phylogenetic relationship between MenFD in algae and plants with homologs from Chlorobi, Gammaproteobacteria, *Rubrobacter xylanophilus*, and *Salinibacter ruber *(BPP = 1.0, PB = 100%, NJB = 100%). These taxa also share a similar operon structure and gene content. Cyanobacterial MenFD in Group 2 is distantly related to the algal/plant proteins forming a sister group to Bacteroidetes and Flavobacteria. The approximately unbiased (AU) test was used to reposition the cyanobacterial clade on all other basal (20 in total) branches in the tree. Using this approach, the union of cyanobacteria with the algal/plant clade in Group 1 was significantly rejected by the AU-test (P < 0.001). Together these results indicate that the phylogenetic history of MenF and MenD proteins is not congruent with a cyanobacterial ancestry and most likely reflects an ancient HGT event.

**Figure 2:**
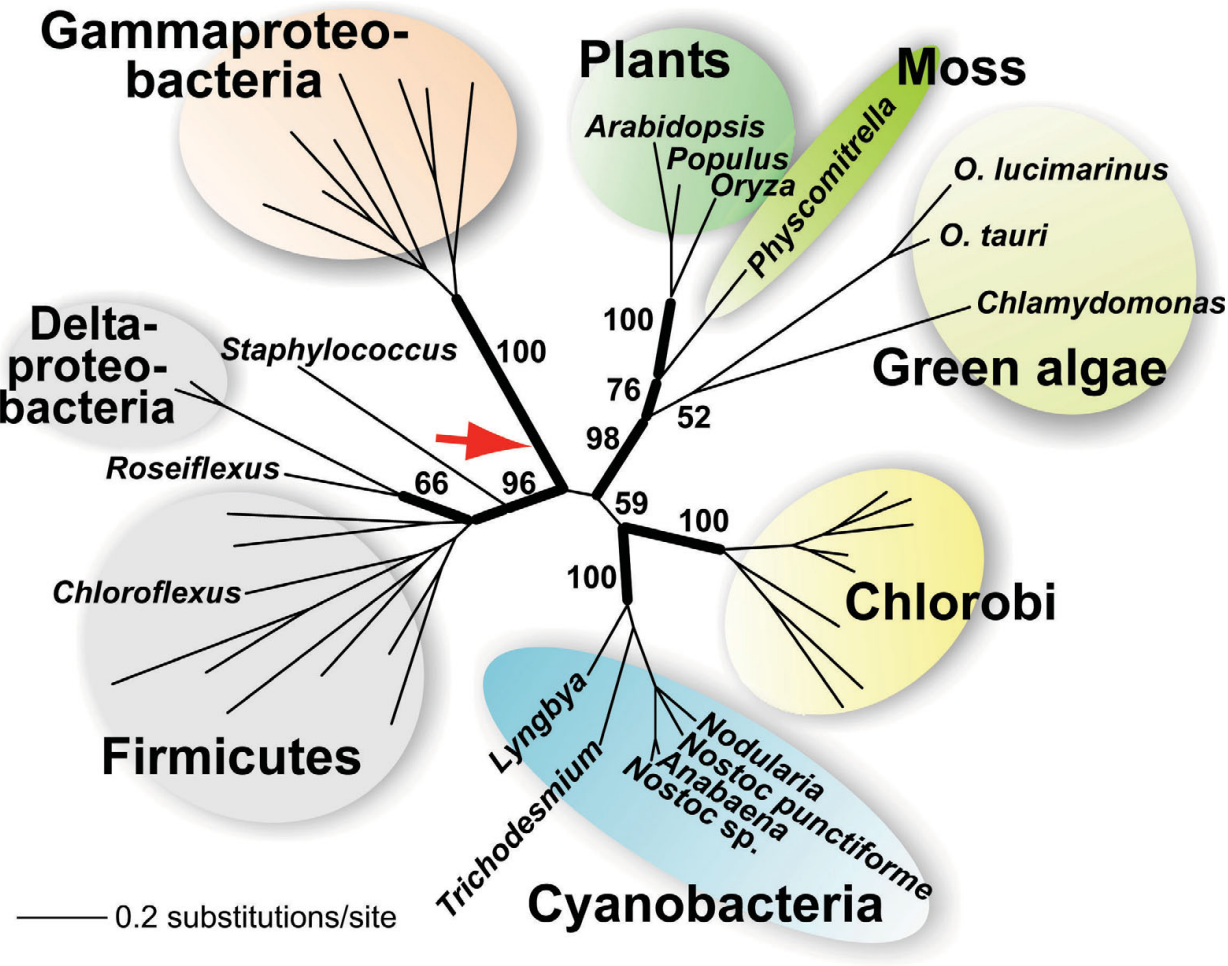
**Unrooted Bayesian majority rule consensus tree of MenH.** Chlorobi and the cyanobacterial groups Oscillatoriales and Nostocales form sister clades, suggesting a HGT between these taxa. The position of diatoms (i.e., *T. pseudonana *and *P. tricornutum*) MenH is indicated by the red arrow. These highly diverged long branched sequences were excluded from the final analysis. The numbers at the nodes are PHYML bootstrap values (500 replicates). Only bootstrap values >50% are shown. For the sake of clarity only support values at nodes of highest interest have been included in the figure. The thick branches have a BPP > 0.95. The branch lengths in this tree are proportional to the number of substitutions per site (see scale in figure).

The topology recovered for the MenC, MenB, MenE, and MenH trees (see Additional files 1, 2, 3) recapitulates the MenFD phylogeny. MenC (part of PHYLLO) and MenB are nuclear-encoded in photosynthetic eukaryotes. These proteins and the homologs encoded in the plastid genome of Cyanidiales are phylogenetically most closely related to sequences in the MQ operon of Chlorobi and Gammaproteobacteria. This is also the case for plastid-encoded MenE in Cyanidiales (see Additional file 3) that is nested in a common clade with homologs in Chlorobi/Gammaproteobacteria. In contrast, MenE is encoded in the nuclear genome of *Arabidopsis *and other plants and algae and branches with Deltaproteobacteria suggesting a phylogenetic origin that is distinct from the Cyanidiales gene (see Additional file 3). Following the trend shown for MenFD (Figure 1b), in the trees of MenC, MenB, and MenE the cyanobacterial homologs are phylogenetically distantly related to the algal/plant clade (see Additional files 1, 2, 3). MenH proteins are almost exclusively found in taxa of Group 1 (for exceptions see below) and the homologs of plants and algae are nested (BPP = 1.0, PB = 96%) among Chlorobi and Gammaproteobacteria homologs (Figure 2). The consistent phylogenetic pattern observed for the six first enzymes of the PhQ biosynthesis in plants and algae (with the exception of the nuclear encoded MenE) suggests that they have the same origin. This implies that the cyanobacterial ancestor of the primary plastid acquired a complete *men *gene cluster (presumably as an operon) via HGT from a single source related to Chlorobi/Gammaproteobacteria. That Men homologs of *Rubrobacter xylanophilus *and *Salinibacter ruber *are also nested within Group 1 representatives and are distantly associated to their taxonomic relatives (Actinobacteria and Bacteroidetes homologs are in the Group 2) suggests that these taxa also gained their *men *operon from a donor related to the source of the algal/plant genes (Figure 1b).

### Independent acquisition of *men*H by cyanobacteria from a source related to Chlorobi

In contrast to the general absence of a detectable *men*H gene from taxa of Group 2 (Figure 1b), we were able to identify this gene in members of the cyanobacterial families Oscillatoriales and Nostocales. In *Trichodesmium erythraeum *(Figure 1b) *men*H is encoded within the *men *gene cluster. Surprisingly, phylogenetic analysis of MenH (Figure 2) demonstrates that cyanobacteria have acquired this gene from Chlorobi-related bacteria. This tree shows that algal and plant MenH is not closely related to cyanobacterial homologs. Therefore, the *men*H genes in Oscillatoriales and Nostocales are not vestiges of the ancient gene cluster that was putatively transferred via primary endosymbiosis into eukaryotes but rather is a more recent independent HGT event in some groups of cyanobacteria. This result illustrates the feasibility of *men *gene transfer into cyanobacteria and supports our model of an ancient gene cluster acquisition by the plastid ancestor.

### The MenA phylogeny supports additional HGT events during plastid evolution

**Figure 3:**
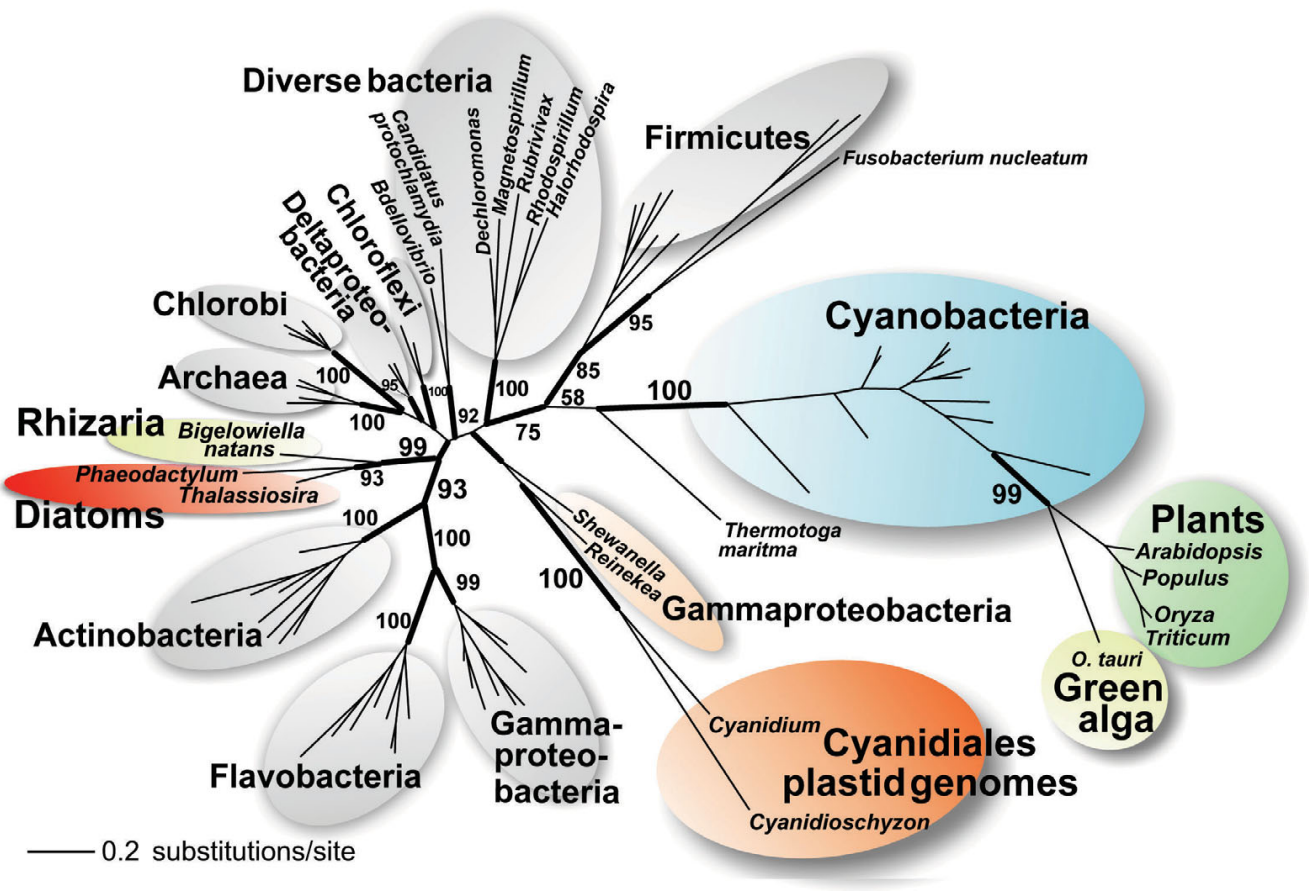
**Unrooted Bayesian majority rule consensus tree of MenA.** Green algae and plants have a cyanobacterial form of MenA, whereas plastid-encoded MenA in Cyanidiales is unrelated to cyanobacteria and likely originated from a HGT event from a distantly related source that introduced (and thereby duplicated) this gene in the genome of the plastid ancestor. The numbers at the branches are PHYML bootstrap values (500 replicates). Only bootstrap values >50% are shown. For the sake of clarity only support values at nodes of highest interest have been included in the figure. The thick branches have a BPP > 0.95. The branch lengths in this tree are proportional to the number of substitutions per site (see scale in figure).

A *men*A gene is also present in the *men *cluster in the plastid genome of Cyanidiales (Figure 1b), whereas it is nuclear encoded in other algae and plants. The phylogeny of MenA reveals however that the Cyanidiales gene is not closely related to homologs in cyanobacteria, Chlorobi, or to the majority of Gammaproteobacteria (Figure 3). Instead, Cyanidiales MenA is monophyletic (BPP = 1.0) with two Gammaproteobacteria (*Shewanella woodyi *and *Reinekea *sp. MED297) that are distantly related to the other members of this bacterial clade. The AU-test in which the Cyanidiales MenA clade was moved to 20 alternate basal branches in the tree significantly rejects the sister group relationship of this clade to cyanobacteria (P = 0.017), Chlorobi (P = 0.009), or Gammaproteobacteria (P = 0.001). These results imply that the *men*A gene and the *men *gene cluster were obtained in different HGT events, involving distinct donor taxa. The *men*A gene has been incorporated into the gene cluster and exclusively maintained in the plastid genome of Cyanidiales with the remainder of the *men *genes. Furthermore, the phylogeny of MenA demonstrates that the homologs encoded in the genome of green algae and plants are of cyanobacterial descent (BPP = 1.0, PB = 100%). One possible explanation of our data is that two *men*A genes were present in the prokaryotic endosymbiont, one of cyanobacterial provenance and other introduced by HGT from another distantly related source. These genes were differentially lost from Cyanidiales and members of the green lineage with the former retaining the plastid-encoded non-cyanobacterial gene and the latter retaining the cyanobacterial copy that was transferred to the nucleus. An alternative explanation is that the *men*A gene in the green lineage was directly acquired from cyanobacteria via HGT into the nucleus of these taxa rather than by EGT from the endosymbiont. Under this scenario, the HGT event was followed by loss of the plastid encoded *men*A gene in the ancestor of green algae. *Men*A is not plastid-encoded in other non-Cyanidiales red algae such as *Gracilaria tenuistitpitata *therefore it is unknown which gene copy is nuclear-encoded in these taxa.

MenA in diatoms and the Rhizarian *Bigelowiella natans *are nuclear-encoded and form a monophyletic group (BPP = 1.0, PB = 99%) that is not phylogenetically closely related to homologs in Cyanidiales or the green lineage (Figure 3). These genes define another HGT event that likely occurred in the nucleus of these taxa involving an unknown bacterial source. Chromalveolates (e.g., diatoms) and Rhizaria have recently been proposed to be monophyletic [24] and the MenA tree is consistent with this idea, showing a unique HGT event shared by these taxa. MenG shows a similar phylogenetic history to MenA, except that MenG is no longer plastid-encoded. This gene is present in the nucleus of all photosynthetic lineages and the phylogeny of MenG provides strong support for a cyanobacterial provenance in the green lineage and an origin in Cyanidiales and diatoms from Deltaproteobacteria (see Additional file 4).

## Discussion

### HGT in the plastid ancestor

We propose that *men*F, *men*D, *men*H, *men*C, *men*B, and *men*E that comprise an operon or a gene cluster, and the *men*A gene was introduced via two independent HGT events into the genome of the cyanobacterial ancestor of primary plastids. The remnants of the *men *gene cluster and *men*A are still present in the plastid genome of the Cyanidiales in a prokaryotic-like gene arrangement. In contrast, the components of the horizontally transferred *men *cluster were moved to the nucleus in the majority of algal lineages. An alternative interpretation of our data is that the *men *genes were introduced into the plastid genome of the Plantae ancestor by a HGT event that postdates primary endosymbiosis but precedes the split of at least the red and green algae (i.e., glaucophyte genes have not yet been described). HGT into plastids is the most likely explanation for the origin of the *rbc*L gene in red and chromalveolate algae, the *rpl36 *gene shared by cryptophytes and haptophytes, and the unique occurrence of the *dnaX *gene in *R. salina *(see above) [9,16,17,25]. We suggest that several lines of (albeit) circumstantial evidence argue against this scenario for origin of the *men *gene cluster. The first is that recent comprehensive phylogenomic analyses of plastid genomes uncovered only the three cases of HGT described above [9,17]. This result, when magnified over the >1 billion years of plastid evolution leads to the conclusion that plastid genomes are generally resistant to HGT [9]. Second, the three known plastid HGT events involve a perfect gene replacement (*rpl36*) [9], a novel single gene insertion (*dnaX*) [17], and the replacement of an intact operon (*rbc*LS) [16]. From our perspective, these mechanisms are unlikely to be the explanation for the introduction of the horizontally transferred *men *gene cluster in the plastid lineage for two reasons. First, the plastid genome is essentially free of large new DNA integrants. And second, a sparse, gene-poor *men *cluster of cyanobacterial origin that putatively existed in the endosymbiont would provide a dispersed and poorly conserved substrate for a single replacement involving homologous recombination with a highly dissimilar DNA segment containing six genes of Chlorobi/Gammaproteobacteria provenance. We propose that it is more likely that the introduction of the original *men *gene cluster occurred in the more dynamic genomes of freeliving cyanobacteria, where foreign genes are commonly co-integrated with cyanophages in chromosomal genomic islands in events not involving homologous recombination and that often introduce redundant alleles that duplicate native cyanobacterial genes [26,27]. Following the original integration we suggest that the native cyanobacterial *men *pathway in the plastid forerunner was lost in favour of the foreign gene cluster. The gain of an additional *men *cluster directly into the newly established organelle, thereby creating allelic redundancy is unlikely giving the known tendency of endosymbionts (e.g., plastids, *Carsonella*) to rapidly streamline their genomes by gene loss [28].

Comparative genomic analyses demonstrate that the acquisition of foreign genes by HGT is common among cyanobacteria [29]. In line with this idea, we provide clear evidence for the ancient acquisition by cyanobacteria of a foreign *men*H from a Chlorobi source (Figure 2). Similarly, our phylogeny of MenC (see Additional file 1) shows that this gene in *Crocosphaera watsoni *and *Cyanothece *sp. was introduced by HGT from a Firmicute source. Together, these examples underline the case we make for *men *gene cluster transfer into the once free-living plastid ancestor. In a parallel example, the thecate amoeba *Paulinella chromatophore *has a plastid-like photosynthetic organelle, the cyanelle, which was also derived from a cyanobacterium. The genomes of the cyanelle and the cyanobacteria *Prochlorococcus *and *Synechococcus *that are the closest relatives of the cyanelle share common components of a carboxysomal operon (including the *rbc*L gene) that is phylogenetically most closely related to Gammaproteobacteria. This implies that the carboxysomal operon was introduced by HGT into the common ancestor of *Prochlorococcus *and *Synechococcus *species and the *Paulinella *cyanelle [30]. This constitutes a recent example of the type of HGT event we postulate occurred for the *men *gene cluster ca. 1.5 billion years ago.

### Relics of endosymbiont genome chimericism may now reside in the nucleus

With regard to interphylum HGT, genome analysis of cyanobacteria suggests that 23% of the tested genes provide conflicting phyletic patterns that include cases of HGTs from donors outside the cyanobacterial clade [29]. In contrast, the majority of phylogenetic studies have found a consistent signal of cyanobacterial ancestry in the plastid genome [e.g., [11,12]]. If HGT was similarly prevalent at the time of endosymbiosis (and there is no obvious reason to think otherwise) then one would expect to find many more examples of plastid genes that are not phylogenetically affiliated with cyanobacteria. The current data suggest however that the contribution of interphylum HGT to the cyanobacterial ancestor of plastids was rare and the *men *gene cluster represents an unlikely survivor.

On the other hand, the apparent absence of additional HGT events in the plastid could reflect a lack of thorough analysis of all genes (not just the phylogenetically conserved set) in all plastid genomes to assign their origins. To assess this possibility, we did an additional phylogenomic analysis in which all of the plastid protein-encoding genes in *Cyanidioschyzon*, the glaucophyte *Cyanophora paradoxa*, and the chlorophyte *Nephroselmis olivacea *were used to query our large bacterial plus eukaryotic protein database. The trees were sorted to identify any clear examples of non-cyanobacterial genes in these plastids [15]. No other cases of interphylum HGT were found (consistent with Rice and Palmer [9] and Khan *et al*., [17]) which leads us to postulate that within the limits of these bioinformatic approaches, the remaining set of plastid genes is of cyanobacterial ancestry.

The absence of endosymbiont chimericism that is found in modern-day plastids may reflect the fact that this organelle encodes only 1/10 (ca. 200 out of 2,500 ancestral genes) of the original cyanobacterial genome. Therefore, the evidence for ancient HGT may have been lost over evolutionary time. Genes preserved in plastids generally have conserved roles in transcription and translation (i.e., informational) or encode subunits of multiprotein complexes involved in photosynthesis [31]. These sequences may be more resistant to HGT than the set of metabolic and house-keeping genes (i.e., operational) that were lost. Operational genes have been demonstrated in cyanobacteria to be relatively more frequently transferred than informational genes, although genes in all functional categories are subject to HGT [29]. Many operational genes once encoded in the cyanobacterial ancestor of plastids have been relocated to the nucleus since primary endosymbiosis [31]. The *men*F, *men*D, *men*C, *men*H, and *men*B genes that are derived from original plastid gene cluster, and possibly *men*A and *men*G (of cyanobacterial origin), followed this path and are now nuclear-encoded in the vast majority of algae and plants. From our perspective, a remarkable exception to this rule is the ancestral *men *gene cluster that was, for unknown reasons, 'frozen' in the plastid genome of a single algal group, the Cyanidiales. Finally, phylogenetic studies of nuclear encoded plastid targeted proteins often show a phylogenetic history that is inconsistent with a cyanobacterial ancestry [32-35]. Although these cases can be interpreted as examples of HGT into the nuclear genome of photosynthetic eukaryotes, which is prevalent in some algae (e.g., chromalveolates) [e.g., [36-38]], our study at least raises the possibility that some may have arisen from a putative chimeric cyanobacterial ancestor and trace their origin to EGT [4,5].

In our phylogenomic analysis we failed to detect significant departure from the cyanobacterial origin of plastid-encoded genes apart from the known cases of *rbc*L [16] and MQ/PhQ. This however should not be interpreted as an absence of HGT in the plastid forerunner. In fact, comparative genomic analyses demonstrate that HGT is common in cyanobacteria, affecting ca. 61% of the genes [29]. These transfers are however largely intraphylum and beyond the limits of detection of our study. Therefore it is entirely possible (and probable) that plastid genomes contain a highly reticulated pattern of gene ancestry that predates primary endosymbiosis. In line with this idea, results of a recent analysis of cyanobacteria-derived genes in the nuclear genome of Plantae [39] showed that in terms of presence/absence and sequence similarity, the highest proportion of these proteins could be traced back to *Nostoc *sp. PCC7120 and *Anabaena variabilis *ATCC29143, identifying them as potential donor lineages. Nonetheless a substantial fraction was found to be more similar to homologs in seven other studied cyanobacteria. These data suggest that even if the plastid ancestor was related to heterocyst-forming *Anabaena*/*Nostoc*, the captured cell also contained genes from a phylogenetically broad spectrum of cyanobacterial lineages presumably as a consequence of ancient intraphylum HGT.

## Conclusion

We asked the question, do plastid genomes still preserve vestiges of HGT that occurred during the free-living phase of the cyanobacterial endosymbiont? For this purpose our phylogenetic study targeted the set of Men proteins responsible for the biosynthesis of MQ in eubacteria and PhQ in cyanobacteria and in photosynthetic eukaryotes. These proteins are encoded in the plastid genome of the red algae Cyanidiales and in the nuclear genome of algae and plants, indicating that they were derived from the cyanobacterial endosymbiont that gave rise to the plastids and later on transferred to the nucleus by EGT. This result clearly contradicts the expectation of a cyanobacterial provenance for the MQ/PhQ pathway in Plantae, showing an affiliation of these sequences to Chlorobi/Gammaproteobacteria (Figures 1b, 2; see Additional files 1, 2, 3). This suggests that the cyanobacterial ancestor of the primary plastid contained a complete *men *gene cluster (*men*F, *men*D, *men*H, *men*B, *men*C, and *men*E) that originated via HGT. In addition, the *men*A gene was also independently derived by HGT in the plastid ancestor from an unknown donor. The *men *gene cluster in Cyanidiales retains this ancestral prokaryotic feature and thus represents a 'living fossil' providing a view into ancient genome chimericism. In contrast, Men homologs are nuclear-encoded in the genomes of green algae, plants, and diatoms and are monophyletic with the plastid-encoded homologs in Cyanidiales. This demonstrates that a chimeric component of the endosymbiont genome was relocated in some lineages from the plastid to the nucleus. The ancestral cyanobacterial lineage that donated the plastid either went extinct or is not yet represented in GenBank because the cyanobacteria in all of our trees analysis are clearly monophyletic and display a sporadic pattern of *men *gene retention typical of Group 2 (Figure 1b). We speculate that the discovery of extant cyanobacteria that contain a *men *operon derived from Chlorobi/Gammaproteobacteria would potentially provide an useful piece of evidence to identify the lineage most closely related to the plastid ancestor.

## Materials and methods

### Sequence data mining

The genes for MQ and PhQ biosynthesis have previously been characterized in *E. coli *and in *Synechocystis *sp. PCC 6803 [19,20], as has *PHYLLO *(with the functional *men*DCH modules), the *men*F genes (*ICS*), *men*G, *men*A, and *men*E in *Arabidopsis *[18,21-23]. The product of all these genes served as queries to find by BLASTP [40] searches the corresponding homologs in other prokaryotes and eukaryotes. For the collection of proteins and initial analysis of the structure of *men *gene clusters, about 120 taxa of menaquinone-using bacteria were selected among the major prokaryotic phyla. Only a fraction of taxa from this original data set was used for phylogenetic analysis. All retrieved Men proteins were authenticated by the presence of the corresponding coding genes within clusters of *men *genes, as well by comparisons to aligned modules of Men proteins in the Conserved Domain Database [41]. This approach allowed us to identify the majority of Men homologs. The use of positive cases also made it possible to deduce cut-offs of the e-value for each family of proteins to identify homolgs by BLASTP in some taxa where Men proteins were not encoded within *men *gene clusters.

The eukaryotic proteins from *Arabidopsis *and rice and the Cyanidiales *Cyanidium caldarium *and *Cyanidioschyzon merolae *were obtained from GenBank [42]. The nucleotide sequence of part of the *men *gene cluster of *Galdieria maxima*, containing *men*B, *men*F, and *men*D, was determined in our laboratory (GenBank accession number EU281345). The remaining plant sequences of *Physcomitrella patens *ssp. *patens, Populus trichocarpa*, the green algae *Chlamydomonas reinhardtii*, *Ostreococcus lucimarinus*, and *Ostreococcus tauri*, and the diatom algae *Phaeodactylum tricornutum*, and *Thalassiosira pseudonana *were retrieved from the DOE joint genome institute database [43]. *PHYLLO *was identified in all these organisms with variations in its modular structure. In green algae and the moss *Physcomitrella patens PHYLLO *displays the architecture *men*FDCH. In higher plants the *men*F enzymatic function is provided by an individual gene (*isochorismate synthase*, *ICS*) that originated via duplication of the *men*F module of *PHYLLO *early in vascular plant evolution [18]. This event was followed by fission of the 3' region of the *PHYLLO men*F module. As a consequence, the architecture of *PHYLLO *(*men*F5'DCH) in higher plants presents a short inactive *men*F module that is homologous to the 5' region of *men*F genes [18]. In diatoms the *men*C module of the *PHYLLO *follows *men*H. In addition, the 3' module encoding a domain of unknown function (*duf*, COG5637) is also combined to the preceding *men *components, resulting in the pentamodular fusion *men *FDHC *duf*.

All of the retrieved sequences were manually annotated with help of the comparative prediction algorithms FGENESH+ [44] and Wise2 [45] combined with multiple alignments with known genes of plant and algae. This approach permitted us to deduce exon-intron boundaries with maximum confidence overcoming common errors resulting from automated annotation pipelines. Sequences were aligned using ClustalW [46] and manually edited. Only conserved regions of proteins that could be unambiguously aligned were used to construct phylogenetic trees. The protein alignments with accession numbers are available from the Debashish Bhattacharya lab web page [47].

### Phylogenetic analysis

The phylogeny of the large alignment of MenFD was inferred using MrBayes [48]. Metropoliscoupled Markov chain Monte Carlo from a random starting tree was used in this analysis with two independent runs (i.e., nrun = 2 command) and 1 cold and 3 heated chains. The Bayesian analysis (aamodel = mixed; rates = invgamma) was run for 2 million generations with trees sampled every 100th generation. To increase the probability of chain convergence, we sampled trees after the standard deviation values of the two runs were < 0.05 to calculate the posterior probabilities. The remaining phylogenies were discarded as burn-in. Bootstrap support for nodes in the majority rule consensus Bayesian tree was determined using maximum likelihood (i.e., PHYML V2.4.3 [49]) and neighbor joining (NJ; MEGA [50]). For the PHYML analysis the model parameters were: model of amino acid substitution = WAG (identified using ProtTest V1.3 [51] with "Fast" optimization and a BIONJ tree); starting tree = NJ; gamma = 1.361; p-inv. = 0.073. For the MEGA analysis: model of amino acid substitution = JTT; gamma = 0.84 (obtained using ProtTest).

The MenH and MenA trees were also inferred using MrBayes as described above. Nodal support was assessed using PHYML bootstrap. The model parameters for these analyses were as follows. MenH PHYML: model of amino acid substitution = WAG (identified using ProtTest); starting tree = NJ; gamma = 2.014; p-inv. = 0.067. MenA PHYML: model of amino acid substitution = Blosum62 (identified using ProtTest); starting tree = NJ; Gamma = 1.591; p-inv. = 0.025. The MenC, MenB, MenE and MenG trees were constructed using MrBayes with specific parameters for each tree as indicated (see Additional files 1, 2, 3, 4).

To address alternative hypotheses about the tree topologies, we generated a backbone phylogeny in each case that was identical to the 'best' PHYML tree but excluded the group of interest. Using this backbone tree, the group of interest was then added individually using MacClade V4.05 [52] to 20 alternative branches that spanned the most likely placements of the clade. The site-by-site likelihoods for the trees in this analysis were calculated using the appropriate data set and model with TREEPUZZLE (V5.2, 31) [53] and the default settings. The approximately unbiased (AU-) test was then implemented using CONSEL V0.1i [54] to assign the tree probabilities.

## List of Abbreviations

AU-test: approximately unbiased test; BPP: Bayesian posterior probability; EGT: endosymbiotic gene transfer; HGT: horizontal gene transfer; *ICS*: *isochorismate synthase*; MQ: menaquinone; NJ: neighbor joining; NJB: neighbor joining bootstrap; PB: PHYML bootstrap; PhQ: phylloquinone.

## Authors' contributions

JG conceived and designed the project, collected and analysed the data and performed the phylogenetic analysis. JM helped to supervise the project. DB helped design the phylogenetic approach, analysed the data, and supervised the project. JG wrote the first draft of the manuscript and DB is responsible for the final version. All authors read and approved the final manuscript.

## Supplementary Material

Additional file 1 Phylogeny of MenC. This figure indicates that the MenC module of PHYLLO and the individual plastidencoded MenC proteins of Cyanidiales are related to homologs of Chlorobi and Gammaproteobacteria. This is a Bayesian majority rule consensus tree using 58 taxa. Posterior probability support values are only indicated (as percentages) for external nodes of the major clades. Analysis parameters: mcmc ngen = 500,000; startingtree = PHYML; samplefreq = 100; aamodel = mixed; rates = invgamma; burnin = 1,250. Click here for file

Additional file 2 Phylogeny of MenB. The tree is juxtaposed to a partial alignment of the corresponding amino acid positions 69 – 116 in the *E. coli *MenB homolog (GenBank accession number, NP_416765.1). Two deletions are correlated with the tree topology. Note that deletion 2 is shared by all Group 1 taxa in Fig. 1b, including photosynthetic eukaryotes, Chlorobi, and Gammaproteobacteria. This is a Bayesian majority rule consensus tree using MrBayes and the following parameters: mcmc ngen = 500,000; startingtree = PHYML; samplefreq = 100; aamodel = mixed; rates = invgamma; burnin = 1,250. Posterior probability support values are only indicated (as percentages) for external nodes of the major clades. Click here for file

Additional file 3 Phylogeny of MenE. The plastid-encoded *men*E of Cyanidiales has its origin in the same gene cluster that was transferred to the plastid ancestor from a Chlorobi/Gammaproteobacteria source. The MenE proteins in the nuclear genome of the remaining algae and plants have a different phylogenetic provenance and are most likely derived by HGT, presumably directly into the host nucleus. This is a Bayesian majority rule consensus tree using 66 taxa. Posterior probability support values are only indicated (as percentages) for external nodes of the major clades. Analysis parameters are: mcmc ngen = 500,000; startingtree = PhyML generated tree; samplefreq = 100; aamodel = mixed; rates = invgamma; burnin = 1,250. Click here for file

Additional file 4 Phylogeny of MenG/UbiE. MenG proteins are responsible for the methylation step of the MQ/PhQ pathway (Lohmann et al. 2006). In eubacteria the same enzymes also catalyse the methylation required for biosynthesis of ubiquinone (Meganathan 2001). In this case MenG homologs are referred to as UbiE. In photosynthetic eukaryotes UbiE functions in mitochondria and MenG is plastid targeted for the synthesis of PhQ. In accordance with its organellar distribution, UbiEs are related to homologs in Alphaproteobacteria, whereas MenG in the nuclear genomes of plants and green algae is derived from the cyanobacterial endosymbiont. MenG encoded in the nuclear genome of Cyanidiales and diatoms are related to Deltaproteobacteria and presumably originated via HGT. This is Bayesian majority rule consensus tree using 81 taxa. Posterior probability support values are only indicated (as percentages) for external nodes of the major clades. Analysis parameters: ngen = 1,100,000; startingtree = PHYML generated tree; samplefreq = 100; aamodel = mixed; rates = gamma; burnin = 2,750. Click here for file
