# Supplementary material for: Evidence of a chimeric genome in the cyanobacterial ancestor of plastids
Source: BMC Evol Biol. 2008 Apr 23;8:117. doi: 10.1186/1471-2148-8-117 (PMC2412073; doi:10.1186/1471-2148-8-117)
Supplement: Additional file 1 — Phylogeny of MenC. This figure indicates that the MenC module of PHYLLO and the individual plastidencoded MenC proteins of Cyanidiales are related to homologs of Chlorobi and Gammaproteobacteria. This is a Bayesian majority rule consensus tree using 58 taxa. Posterior probability support values are only indicated (as percentages) for external nodes of the major clades. Analysis parameters: mcmc ngen = 500,000; startingtree = PHYML; samplefreq = 100; aamodel = mixed; rates = invgamma; burnin = 1,250. [file 1471-2148-8-117-S1.pdf]

Additional files:

Evidence of a chimeric genome in the cyanobacterial ancestor of plastids

Jeferson Gross[1], Jörg Meurer[2], and Debashish Bhattacharya[1]

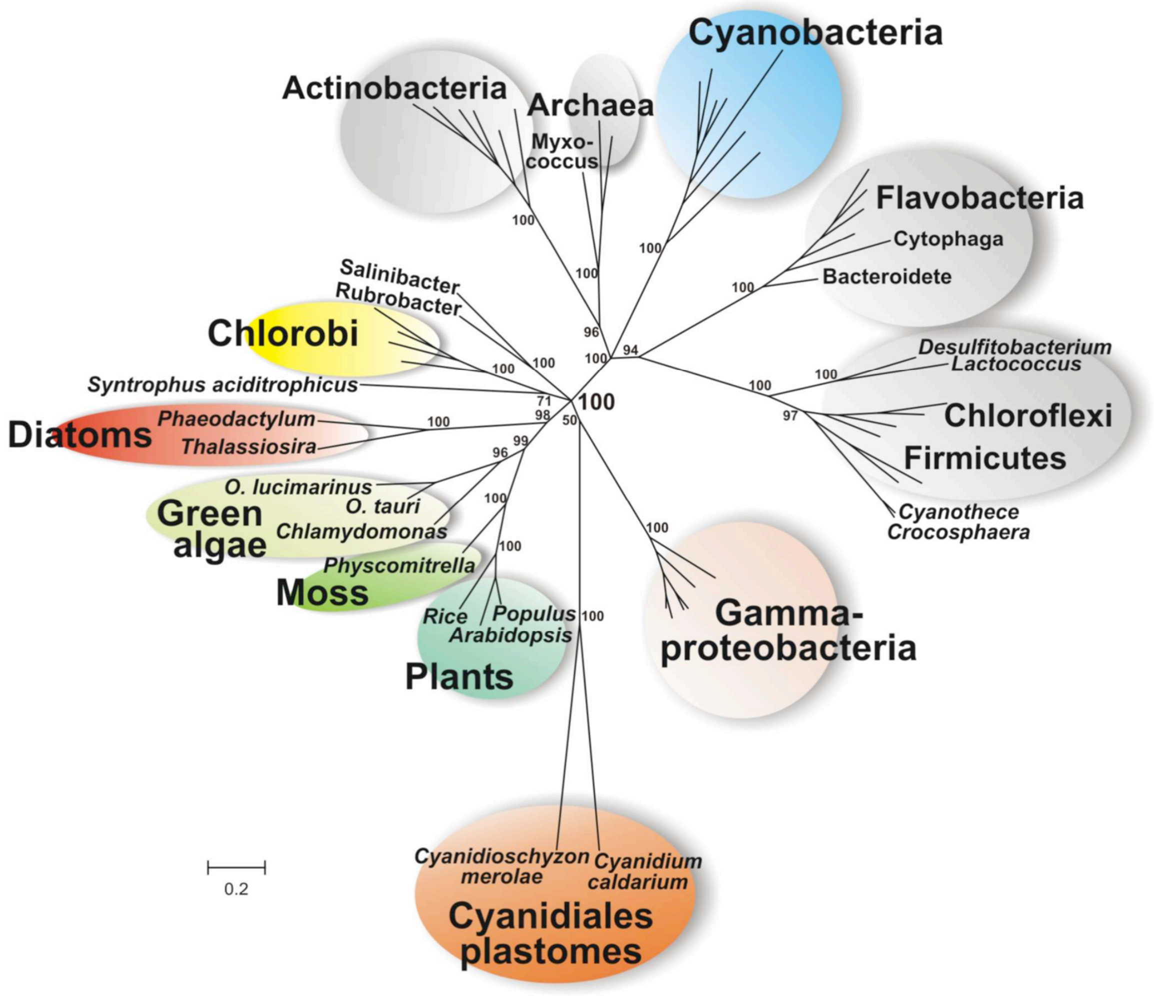

Additional file 1. Phylogeny of MenC. This figure indicates that the MenC module of PHYLLO and the individual plastid-encoded MenC proteins of Cyanidiales are related to homologs of Chlorobi and Gammaproteobacteria. This is a Bayesian majority rule consensus tree using 58 taxa. Posterior probability support values are only indicated (as percentages) for external nodes of the major clades. Analysis parameters: mcmc ngen=500,000; startingtree=PHYML; samplefreq=100; aamodel=mixed; rates=invgamma; burnin=1,250.
