# Supplementary material for: Evidence of a chimeric genome in the cyanobacterial ancestor of plastids
Source: BMC Evol Biol. 2008 Apr 23;8:117. doi: 10.1186/1471-2148-8-117 (PMC2412073; doi:10.1186/1471-2148-8-117)
Supplement: Additional file 2 — Phylogeny of MenB. The tree is juxtaposed to a partial alignment of the corresponding amino acid positions 69 – 116 in the E. coli MenB homolog (GenBank accession number, NP_416765.1). Two deletions are correlated with the tree topology. Note that deletion 2 is shared by all Group 1 taxa in Fig. 1b, including photosynthetic eukaryotes, Chlorobi, and Gammaproteobacteria. This is a Bayesian majority rule consensus tree using MrBayes and the following parameters: mcmc ngen = 500,000; startingtree = PHYML; samplefreq = 100; aamodel = mixed; rates = invgamma; burnin = 1,250. Posterior probability support values are only indicated (as percentages) for external nodes of the major clades. [file 1471-2148-8-117-S2.pdf]

Additional files:

Evidence of a chimeric genome in the cyanobacterial ancestor of plastids

Jeferson Gross[1], Jörg Meurer[2], and Debashish Bhattacharya[1]

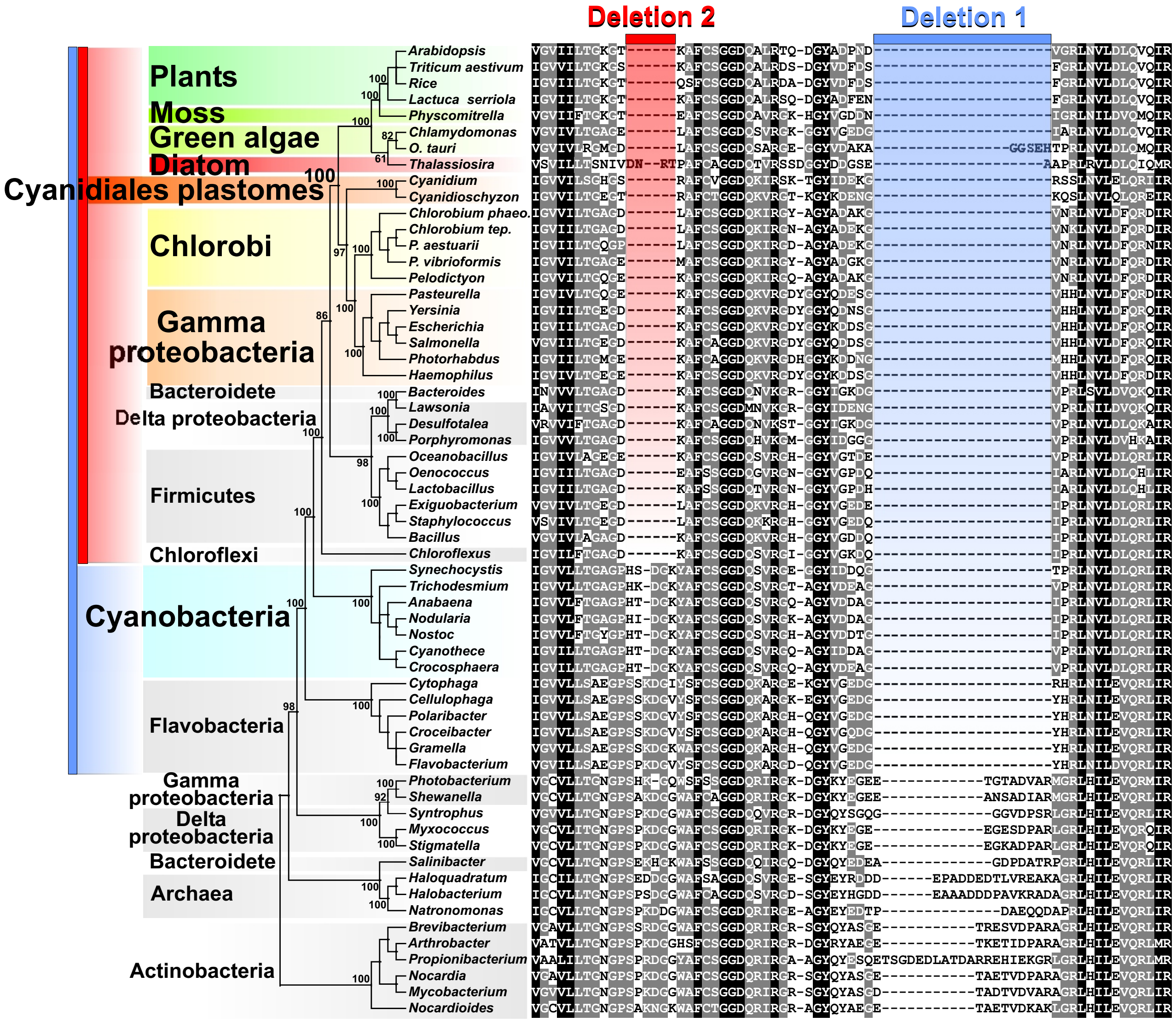

Additional file 2. Phylogeny of MenB. The tree is juxtaposed to a partial alignment of the corresponding amino acid positions 69 – 116 in the *E.coli* MenB homolog (GenBank accession number, NP_416765.1). Two deletions are correlated with the tree topology. Note that deletion 2 is shared by all Group 1 taxa in Fig. 1b, including photosynthetic eukaryotes, Chlorobi, and Gammaproteobacteria. This is a Bayesian majority rule consensus tree using MrBayes and the following parameters: mcmc ngen=500,000; startingtree=PHYML; samplefreq=100; aamodel=mixed; rates=invgamma; burnin=1,250. Posterior probability support values are only indicated (as percentages) for external nodes of the major clades.
