# Supplementary material for: Evidence of a chimeric genome in the cyanobacterial ancestor of plastids
Source: BMC Evol Biol. 2008 Apr 23;8:117. doi: 10.1186/1471-2148-8-117 (PMC2412073; doi:10.1186/1471-2148-8-117)
Supplement: Additional file 3 — Phylogeny of MenE. The plastid-encoded menE of Cyanidiales has its origin in the same gene cluster that was transferred to the plastid ancestor from a Chlorobi/Gammaproteobacteria source. The MenE proteins in the nuclear genome of the remaining algae and plants have a different phylogenetic provenance and are most likely derived by HGT, presumably directly into the host nucleus. This is a Bayesian majority rule consensus tree using 66 taxa. Posterior probability support values are only indicated (as percentages) for external nodes of the major clades. Analysis parameters are: mcmc ngen = 500,000; startingtree = PhyML generated tree; samplefreq = 100; aamodel = mixed; rates = invgamma; burnin = 1,250. [file 1471-2148-8-117-S3.pdf]

Additional files:

Evidence of a chimeric genome in the cyanobacterial ancestor of plastids

Jeferson Gross[1], Jörg Meurer[2], and Debashish Bhattacharya[1]

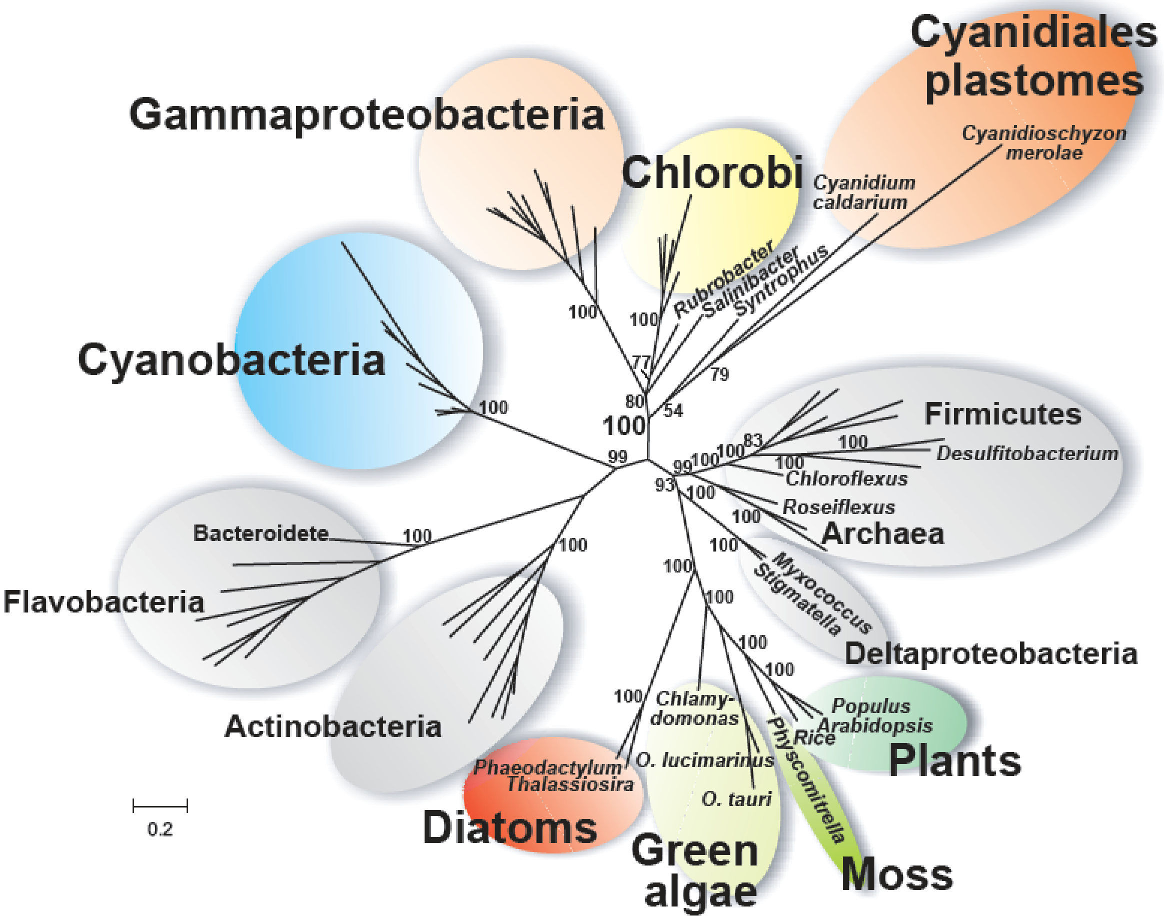

Additional file 3. Phylogeny of MenE. The plastid-encoded *men*E of Cyanidiales has its origin in the same gene cluster that was transferred to the plastid ancestor from a Chlorobi/Gammaproteobacteria source. The MenE proteins in the nuclear genome of the remaining algae and plants have a different phylogenetic provenance and are most likely derived by HGT, presumably directly into the host nucleus. This is a Bayesian majority rule consensus tree using 66 taxa. Posterior probability support values are only indicated (as percentages) for external nodes of the major clades. Analysis parameters are: mcmc ngen=500,000; startingtree=PhyML generated tree; samplefreq=100; aamodel=mixed; rates=invgamma; burnin=1,250.
