# Supplementary material for: Evidence of a chimeric genome in the cyanobacterial ancestor of plastids
Source: BMC Evol Biol. 2008 Apr 23;8:117. doi: 10.1186/1471-2148-8-117 (PMC2412073; doi:10.1186/1471-2148-8-117)
Supplement: Additional file 4 — Phylogeny of MenG/UbiE. MenG proteins are responsible for the methylation step of the MQ/PhQ pathway (Lohmann et al. 2006). In eubacteria the same enzymes also catalyse the methylation required for biosynthesis of ubiquinone (Meganathan 2001). In this case MenG homologs are referred to as UbiE. In photosynthetic eukaryotes UbiE functions in mitochondria and MenG is plastid targeted for the synthesis of PhQ. In accordance with its organellar distribution, UbiEs are related to homologs in Alphaproteobacteria, whereas MenG in the nuclear genomes of plants and green algae is derived from the cyanobacterial endosymbiont. MenG encoded in the nuclear genome of Cyanidiales and diatoms are related to Deltaproteobacteria and presumably originated via HGT. This is Bayesian majority rule consensus tree using 81 taxa. Posterior probability support values are only indicated (as percentages) for external nodes of the major clades. Analysis parameters: ngen = 1,100,000; startingtree = PHYML generated tree; samplefreq = 100; aamodel = mixed; rates = gamma; burnin = 2,750. [file 1471-2148-8-117-S4.pdf]

Additional files:

Evidence of a chimeric genome in the cyanobacterial ancestor of plastids

Jeferson Gross[1], Jörg Meurer[2], and Debashish Bhattacharya[1]

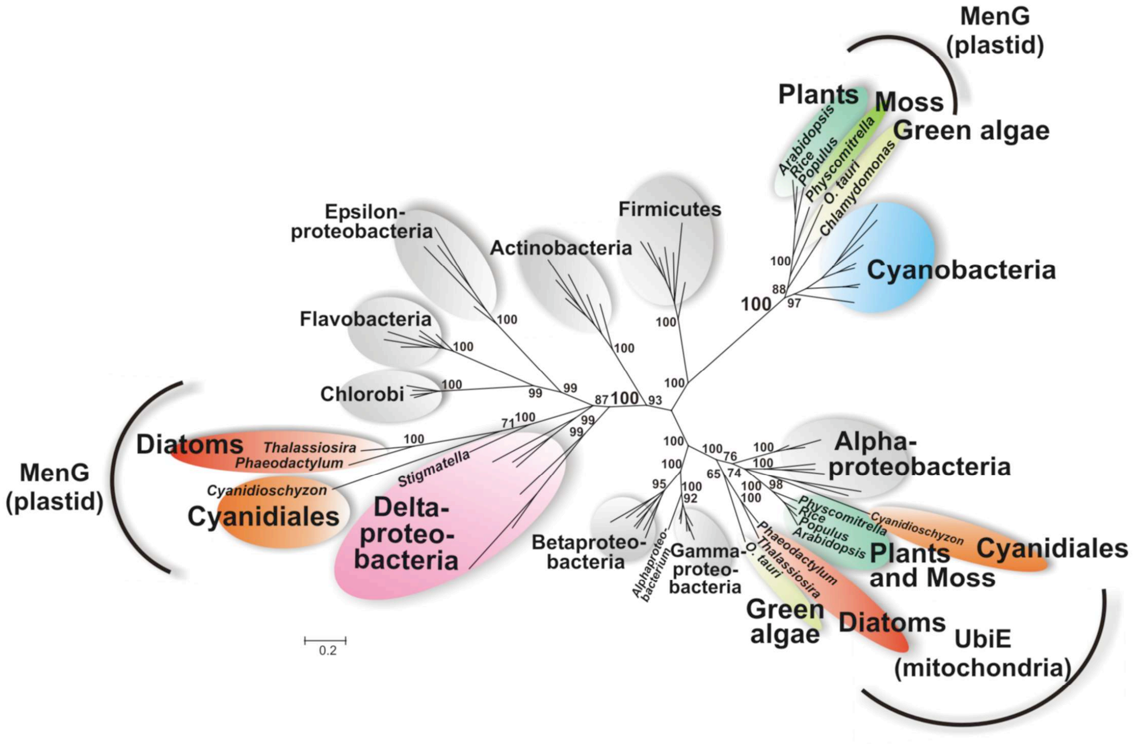

Additional file 4. Phylogeny of MenG/UbiE. MenG proteins are responsible for the methylation step of the MQ/PhQ pathway (Lohmann et al. 2006). In eubacteria the same enzymes also catalyse the methylation required for biosynthesis of ubiquinone (Meganathan 2001). In this case MenG homologs are referred to as UbiE. In photosynthetic eukaryotes UbiE functions in mitochondria and MenG is plastid targeted for the synthesis of PhQ. In accordance with its organellar distribution, UbiEs are related to homologs in Alphaproteobacteria, whereas MenG in the nuclear genomes of plants and green algae is derived from the cyanobacterial endosymbiont. MenG encoded in the nuclear genome of Cyanidiales and diatoms are related to Deltaproteobacteria and presumably originated via HGT. This is Bayesian majority rule consensus tree using 81 taxa. Posterior probability support values are only indicated (as percentages) for external nodes of the major clades. Analysis parameters: ngen=1,100,000; startingtree=PHYML generated tree; samplefreq=100; aamodel=mixed; rates=gamma; burnin=2,750.
